# Study on *Periplaneta americana* (Blattodea: Blattidae) Fungal Infections in Hospital Sewer System, Esfahan City, Iran, 2017

**DOI:** 10.1155/2020/4296720

**Published:** 2020-07-26

**Authors:** Maryam Khodabandeh, Leila Shirani-Bidabadi, Mahboobe Madani, Alireza Zahraei-Ramazani

**Affiliations:** ^1^Islamic Azad University Falavarjan Branch, Faculty of Microbiology, Department of Microbiology, University Boulevard, Post Box: 155/84515, Falavarjan, Iran; ^2^Kerman University of Medical Sciences, School of Public Health, Department of Vector Biology and Control of Disease, Haft-Bagh Highway, Post Box: 76169-13555, Kerman, Iran; ^3^Tehran University of Medical Sciences, School of Public Health, Department of Medical Entomology and Vector Control, Enghelab Street, Post Box: 6446-14155, Tehran, Iran

## Abstract

**Background:**

American cockroaches contaminated with pathogens inside hospital manholes can be one of the major problems that health care systems face.

**Objectives:**

The aim of this study was to investigate the fungal infections of American cockroaches in the Esfahan hospital sewage network. The principle goal of the study was about the roaches as a vector of fungi and other pathogens.

**Method:**

The type of study was descriptive-analytical. A total of 55 American cockroach specimens from the manhole walls of the sewerage system of 7 large hospitals were captured. Samples were taken from the surface of the body, digestive tract, and haemocoel of cockroaches. The specimens were then cultured on Sabouraud dextrose agar separately, and fungi were identified according to the macroscopic and microscopic characteristics.

**Results:**

All cockroaches collected from hospitals were infected with fungi. Among the 24 (13 infected and 11 noninfected) (44%) female cockroaches and 31 (18 infected and 13 noninfected) (56%) male cockroaches, it was identified that 40.00% was infected with *Aspergillus niger*, 3.64% with *Rhizopus*, 7.27% with *Penicillium*, and 5.45% with *Mucor*. 6 cockroaches had no yeast contamination. 17 (30.91%) cockroaches were contaminated with *Candida glabrata*, 23 (41.82%) cockroaches were contaminated with *Candida krusei*, and *22* (40%) cockroaches were contaminated with other yeast species. The results of this study showed that *Candida krusei* had the highest prevalence among the isolated fungi with 35.37% of the digestive system and *Aspergillus niger* with 70.97% of the surface of the cockroach body.

**Conclusion:**

The results emphasized the role played by cockroaches as potential pathogenic vectors in hospital environments. Therefore, suitable management is needed for controlling this insect to prevent disease transmission in hospitals.

## 1. Introduction

Cockroaches are one of the oldest inhabitants of the Earth, dating back as far as the Carboniferous period, over 250 million yrs ago [1]. They lack special adaptations like the sucking mouthparts in some insects such as the aphids and other true bugs [2]. Over 4500 species of cockroaches have been identified, of which 40 species are associated with human habitats, while four species are well known as pests [3–5]. Cockroaches have chewing mouthparts and feed on a variety of materials (omnivorous), aiding in the mechanical transmission of various pathogenic viruses, bacteria, and protozoans to humans. Cockroaches have a worldwide distribution, especially in tropical and subtropical areas, and can tolerate a wide range of environments from the Arctic cold to tropical heat [4, 5]. Their population increases in hot and humid places, especially with the availability of food and water [6].

Cockroaches feed indiscriminately on garbage and sewage and so have copious opportunities to disseminate human pathogens [7, 8]. Also, their nocturnal and filthy habits make them ideal carriers of various pathogenic microorganisms [2].

*Periplaneta americana* (Linneaus, 1785) (Blattodea: Blattidae) are cosmopolitan cockroaches that occur frequently in urban sewage galleries and transit in anthropic environments, spreading pathogens; they are considered hazardous organisms to humans, causing serious health problems such as allergies, asthma, and others [9–12].

*Periplaneta americana* is the largest species of cockroaches found in Iran. It originated from South America, measures 30–40 mm in length, and is reddish-brown in color. The American cockroach lives in hot areas of buildings like the kitchens, heating rooms, warehouses, and sewage systems [4]. It usually comes out of its hiding places at night for feeding and other activities [13]. The adult cockroaches are long-lived and can live for as long as one year or more, producing a large number of egg capsules during this period, depending on food availability [14].

Sewerage is established to collect and convey sewage in urban areas in order to prevent contamination of soil and water resources. Some species of cockroaches including *P. americana* have colonized the sewerages, turning them into a suitable environment for reproduction and growth, posing serious health problems to humans [15].

Cockroaches are able to transfer fungi, bacteria, viruses, parasites, and other medically significant pathogenic agents on their body surfaces and in their feces in infectious regions, such as domestic habitats, hospitals, and industrial areas. From these insects collected from such environments, important pathogenic microorganisms have been isolated [13–18]. Plentiful pathogenic agents including 2 species of protozoans, 15 species of molds and fungi, 32 species of bacteria (such as *Shigella* and *Salmonella*), 1 virus, and 7 helminths which cause damage to humans are detected in the feces, in the gut, or on the cuticle of the cockroaches [19–21].

Hospitals, especially those with infectious diseases, have become the main areas for the transmission and spread of infections and a good place to grow and reproduce cockroaches, so that they can mechanically transmit those infectious agents and cause various diseases. Therefore, their struggle and control to create a safe and secure environment will achieve the basic goals of the hospitals. American cockroaches, due to specific biological behaviors, cause a variety of nosocomial and fungal infections at the hospital level and transmit and disperse these microorganisms in the environment and in the human places. Seven (external and internal) were found in the sewage network of seven hospitals with an infectious ward in Esfahan. The objective of our study was to investigate the fungal infections of American cockroaches in the Esfahan hospital sewage network.

## 2. Materials and Methods

### 2.1. Study Area

**Figure 1:**
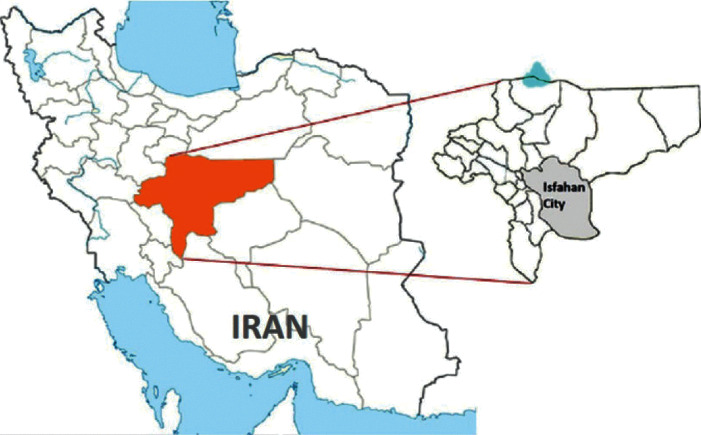
Study area of the Esfahan state and the city located in the central part of Iran, 2017 (prepared by the authors).

This research was carried out in the Esfahan city (51°39′40″E 32°38′30″N) as a part of the central coordination of Esfahan, a central province of Iran. Esfahan is the capital city of the Esfahan province with an area of 250 km^2^ and 1695789 residents. It is the second most populous metropolitan city after Tehran in the central of Iran. Its altitude is 1571 m, latitude 32°38′30″N, and longitude 51°39′40″E (Figure 1). This city is located in a dry semidesert region with an annual rainfall of 102 mm and average summer maximum and winter minimum temperatures of 37.1°C and −5.81°C, respectively [22]. The Esfahan city has over 55375 manholes in its sewage system with an average depth of 9 m on the main lines and 1 meter on lateral lines. Most of the manhole covers were cast irons with two holes, and the average diameter of the holes was three centimeters. The shafts of the manholes were made of brick and cement coverings, and their stable moderate warmth and high humidity provide excellent conditions for cockroaches [23].

### 2.2. Collection Methods of Cockroaches

The sampling strategy of this study was the randomly clustered method from the internal sewage network manholes. Based on the sample size determination formula *n* = *pqz*^2^/*d*^2^*z* = 1.96, *p* = 70% *q* = 1 − *p* = 30% *d* = 0.2, the sample size calculated was ∼21 samples. In this descriptive-analytical study, 55 American cockroaches randomly clustered were sampled from the internal sewage network manholes of 7 hospitals out of the 27 active hospitals in the urban area of the city of Esfahan, Iran, during 2016–2017, which necessarily had a large number of infectious, hospitalized, and traffic units. If there were not enough cockroaches in the first manhole, the second manhole was used. These 7 hospitals have been selected in such a way that their distribution indicates the entire city of Esfahan, i.e., they were selected in the north, south, east, and west, as well as the center of Esfahan.

Direct collection (hand capture) was used to collect the cockroaches. Cockroaches infected with white mycelium were slow-moving. The cockroaches were collected without being harmed. The name of the hospital and the exact location of the sample were noted on a label affixed to the containers. White moldy cockroaches were slower than other cockroaches. Each cockroach was placed in a single sterile test tube (falcon tube 15 ml), which was sterilized before, and transported to the laboratory at the Esfahan Health Research and Training Center, Tehran University of Medical Sciences, for identification and processing for fungi examination.

### 2.3. Isolation of Fungi from Cockroaches

In the laboratory, the cockroaches were immobilized by freezing at 0°C for 10 min. Each anesthetized cockroach was examined under the dissecting microscope, and the species were identified using standard taxonomic keys. After identification, 2 ml of sterile normal saline (0.9% NaCl salt) was added to the test tube, and the cockroaches were vigorously shaken for 2 min. After external washing, the cockroaches were washed with 70% ethyl alcohol for 2 min. Then, the cockroaches were transferred to sterilized tubes and allowed to dry. Then, each cockroach was anesthetized with a chloroform-impregnated cotton pad and fixed on a work paper. The legs of the cockroach were fixed with a sterile needle to the work paper, and with the insect surgical scissors, the cockroaches were returned to the back of the body. The cockroaches were then washed twice in sterile normal saline for 3 min to remove traces of alcohol, and the gut was dissected out aseptically. The gut was then macerated under aseptic conditions in 2 ml of sterile normal saline. The haemocoel portion of each cockroach was first homogenized, and the resulting mixture was put in a sterile container, and 2 ml of physiological serum was added to it. The gastrointestinal tract and cockroach homogenate were individually placed in a microtube and homogenized by the vertex apparatus [24, 25].

### 2.4. Mycological Studies

The resulting macerate was cultured on Sabouraud's dextrose agar with 0.05% chloramphenicol and incubated at 37°C for 2 weeks. Suspensions from each cockroach section were centrifuged at 2000 rpm for 10 minutes to perform mycological studies. Then, the outer and inner portions of the precipitate were cultured in 1 ml in three separate plates containing Sabouraud dextrose agar with chloramphenicol. Two culture media were prepared from each sample, one incubated at 25°C and the other at 37°C. Plates were kept for 7–14 days, and during this period, they were examined for fungal growth. The samples were then purified on the culture medium, and microscopic identification of the most frequent fungi was performed. Samples grown on Sabouraud dextrose agar were then purified. To identify different types of fungi, the macroscopic properties of fungal colonies, such as surface color and back of the colonies, colonial surface view of wrinkles, radial lines or concentric circles, and smoothness or creasing of the colonies, as well as the surface conditions of the colonies, such as powdery mildew, cotton, wool, velvety, and specificity, as well as microscopic characteristics were studied [26].

In order to identify yeasts, in addition to their appearance and colony characteristics that are of particular importance for diagnosis, their physiological properties, which are more important for their identification and determination, were used. To this end, the tubal mass or germ tube test and the Corn Meal Agar test were performed to investigate chlamydoconidia, as well as the *Candida* CHROMagar test [26].

### 2.5. Tubal Mass or Germ Tube Test

For *C. albicans*, the germ tube test was performed to differentiate between *C. albicans* and nonalbicans *Candida* (NAC). By using a sterile needle, one colony of pure yeast was added separately to the sterile tube containing 1 ml of fresh human serum. The suspension was stored at 2°C for 1 to 2 hours. Then, a drop of the above suspension was examined under a microscope for the presence or absence of the germ tube. The formation of the germ tube more than twice the diameter of the yeast cell, the parallel wall, and the absence of troughs at the site of the germ tube formation confirm the presence of the yeast *Candida albicans* [27].

### 2.6. Chlamydoconidia Test on Corn Meal Agar with Tween 80 Culture

The colonies from CHROMagar and SDA were plated on the Corn Meal Agar with Tween 80 for morphological examination of the production of chlamydospores, blastospores, true hyphae, and branched pseudohyphae [27].

A linear culture was performed by using a sterile loop of each yeast colonies on the Corn Meal Agar medium containing 1% Tween 80. The media were kept at 30°C for 72 hours and examined directly under a microscope at 24, 48, and 72 hours. Under these conditions, *Candida albicans* produces false hyphae, blastoconidia, and chlamydoconidia. Other species of *Candida* do not cause chlamydiosis [27].

### 2.7. Culture on CHROMagar *Candida* Medium

The CHROMagar *Candida* medium comprised peptone (10 g), glucose (20 g), agar (15 g), chloramphenicol (0.5 g), and chromogenic ix (2 g) per liter, pH 6.1. This medium was prepared according to the manufacturer's instructions and does not require autoclaving and is dispensed into the Petri plates after cooling. The culture was inoculated, and incubation was done at 37°C. The appearance of colonies, including color, size, and textures on the CHROMagar *Candida* medium, was analyzed [27].

All yeasts isolated from cockroaches were cultured on a CHROMagar company medium (CHROMagar company, Paris, France) and kept at 35°C for 48 hours. Based on the specific color created in the culture medium, species identification was performed. This medium is valid for the identification of the yeasts of *Candida albicans*, *Candida tropicalis*, and *Candida krusei*. These yeasts in this medium create a colony of green, gray, and pink colors. The culture on the chromium agar medium of *Candida* is valuable because of its ease of use, its ability to isolate *Candida* from clinical specimens, and the ability to identify up to several *Candida* species in the clinical specimen at a time [27].

Potential limitation of this study included the following: (1) collection of cockroaches from manholes of sewage, (2) isolation and culture of fungi, and (3) the PCR method due to high prices and shortage of materials due to sanctions in Iran.

## 3. Results

A total of 55 American cockroaches, *Periplaneta americana*, were sampled and studied from the sewage manholes of studied hospitals in Esfahan. Among them were 24 (13 infected and 11 noninfected) (44%) female cockroaches and 31 (18 infected and 13 noninfected) (56%) male cockroaches. The results of the cumulative frequency distribution of different fungal species isolated on the basis of fungal isolation from 55 American cockroaches are presented in Table 1.

All cockroach samples were infected with 8 different fungal species. 35.37% of the contamination was related to *Candida krusei and* 70.97% to *Aspergillus niger*. Both mold and yeast were isolated from American cockroaches. Out of the 31 male cockroaches, 15 were infected with *Aspergillus niger*, 1 with *Penicillium*, 1 with *Rhizopus,* 1 with *Mucor*, and 13 with no infection. Out of the 24 female cockroaches, 7 were infected with Aspergillus *niger*, 3 with *Penicillium*, 1 with *Rhizopus*, 2 with *Mucor*, and 11 with no infection.

In relation to yeast fungi in male cockroaches, 10 cases of *Candida glabrata* infection, 15 cases of *Candida krusei* infection, 16 cases of *Candida kluyveri* infection, 5 cases of *Candida viswanathii* infection, and 5 cases of no contamination were reported.

Concerning the female sex of cockroaches, 11 cases of *Candida glabrata* infection, 14 cases of *Candida kudriavzevii* infection, 8 cases of *Candida kluyveri* infection, 3 cases of *Candida viswanathii* infection, and 2 cases of noncontamination were reported (Table 2). There was no significant difference in the abundance of yeast fungi in male and female cockroaches, such as mold fungi.

Based on macroscopic and microscopic observations, 24 cockroaches (43.63%) were not infected with mold, 22 (40%) were infected with *Aspergillus niger*, 2 (3.64%) were infected with *Rhizopus*, 4 (7.27%) were infected with *Penicillium*, and 3 (5.45%) were infected with *Mucor*.

None of the yeasts isolated from cockroaches were *Candida albicans*, according to the results of germ tube testing, Corn Meal Agar, and chromium agar. Based on the specific color created in the chromium agar *Candida* medium, the identification of yeast species was performed. Colonies were created in green, gray, and pink, confirming the yeasts of *Candida albicans*, *Candida tropicalis*, and *Candida krusei* [26]. Large pink-to-purple yeast colonies of *Candida glabrata* were reported [27].

The results of the frequency distribution of yeast fungi isolated from cockroaches showed that 6 cockroaches had no yeast fungal contamination. Also, 17 (30.91%) were infected with *Candida glabrata*, 23 (41.82) with *Candida kudriavzevii*, and 22 (40%) were contaminated with other mold (Table 2).

## 4. Discussion

The main objective of this study was to examine human pathogenic microorganisms (bacteria and fungi) on the external and internal body parts of the American cockroach *(Periplaneta americana)* from the hospital sewer system in Esfahan city, Iran. American cockroach was collected from different manholes of the sewer system. This study showed that American cockroach is one of the dominant and active cockroaches in Esfahan health centers.

Different pathogenic and nonpathogenic fungal agents were recovered from this cockroach in human environments. *Periplaneta americana* can carry pathogenic fungi in its internal organs. Therefore, *P. americana* is much more than a harassment, and it has important health hazards as a mechanical vector. Therefore, the abundance of American cockroaches' population has to be reduced by various control methods, such as the proper management of garbage and organic waste disposal, sanitation, and using safe insecticides. Clearly, the presence of cockroaches in sensitive environments, hospitals, and houses is more dangerous than other parts due to the special circumstances and the special people hospitalized and can affect the environment, people, and community health. The density of cockroaches in most parts of the hospital and residential dwellings, their feeding from secretions and human feces, and their ability to transmit a wide range of pathogenic agents make them an ideal vector to transmit most medically important microorganisms. Nowadays, cockroaches have access to an infection source, human food, and the place for food production, and their role in the transmission of the disease is undeniable. Dehghani et al. studied cockroach fauna and frequency in human residential habitats in the north of Esfahan, in the Shahin Shahr city [28]. 675 of 1000 studied houses (67.5%) were infested by all life stages of the cockroaches, and 32.5% had no infestation. 46% of infested houses had few, 30% had medium, and 24% had high infestation. The bathrooms and toilets were recognized as the most infested places (41%) [28]. The propensity of American cockroaches to move freely and dwell in sewers, restrooms, and drains make the problem worse. The ability of cockroaches in the transmission of pathogens is emphasized in many types of research in this regard. Infectious agents carried by cockroaches can infect humans, animals, and food resources in some conditions [29, 30]. This study confirmed that these insects in residential areas were contaminated with fungi of medical importance. A total of 8 different fungal species were isolated from American cockroaches. In this study, a high number of the cockroach specimens (55 number) from the sewer system were found to carry known fungal pathogens including *Rhizopus* sp., *Mucor* sp*., Penicillium* spp., *Candida* spp., and *Aspergillus* spp. Thus, the isolation of medically important fungi suggests a serious risk concern for patients. Although the direct involvement of American cockroaches in the transmission of infectious agents is difficult to demonstrate, several other studies have also isolated, from cockroaches from residential areas and hospitals, medically important fungi [24, 31, 32].

In the present study, all cockroach samples were infected with 8 different fungal species. 35.37% of the contamination was related to *Candida krusei and* 70.97% to *Aspergillus niger*. Both mold and yeast were isolated from American cockroaches. Out of the 31 male cockroaches, 15 were infected with *Aspergillus niger*, 1 with *Penicillium*, 1 with *Rhizopus,* 1 with *Mucor*, and 13 with no infection. Out of the 24 female cockroaches, 7 were infected with *Aspergillus niger*, 3 with *Penicillium*, 1 with *Rhizopus*, 2 with *Mucor*, and 11 with no infection. The findings from this study about medically important fungi isolated from cockroaches agree with the results of some researchers. In a study in Thailand, *Penicillium* spp. and *Aspergillus* spp. appeared frequently on the integument of 16 (35.6%) and 11 (24.4%) cockroaches, respectively [32]. In another study in Brazil, *Candida* sp. (38.6%), *Aspergillus* sp. (30.7%), and *Penicillium* sp. (8.9%) were the most common fungi recovered on cockroaches [31]. In addition, in Sari (Iran), *Candida* spp., *Aspergillus* spp., and *Rhodotorula* spp. were the most common fungi that appeared on the cuticle of cockroaches [30]. Kasseri et al. [33] in the Khuzestan province, Ahvaz city, showed the fungi isolated from the digestive system of *Periplaneta americana* (Blattaria: Blattidae) trapped from residential dwellings [33]. Results of their study showed that a high percentage of cockroaches (88.6%) were detected to carry fungi of medical importance. Overall, 23 fungi species/genera were isolated from the American cockroaches' alimentary tract. The fungi isolated from cockroaches from the residential regions were species of *Aspergillus*, *Rhizopus*, *Penicillium*, *Mucorales*, *Alternaria*, *Cladosporium*, *Mycelia*, *Chrysosporium*, *Candida*, *Rhodotorula*, *Zygosaccharomyces*, and *Debaryomyces*. *Candida* spp. (41.4%), *Aspergillus* spp. (37.1%), and *Rhodotorula* spp. (27.1%) were the most common fungi recovered on cockroaches. *Candida albicans* and *Candida glabrata* were the commonest species of the genus *Candida*. In addition, *Aspergillus niger* and *A. flavus* were the most frequent species of the genus *Aspergillus* [33].

In a survey in Kashan (Iran) by Droodgar in 2004, the prevalence of fungal agents in cockroaches was 41.1% and 22.8%, respectively. *Candida* spp. (39.5%), *Aspergillus* spp. (37. 2%), and *Penicillium* spp. (5.4%) had a maximum prevalence among the fungi observed [34]. In India, *Candida* spp. and *Aspergillus* spp. were the most frequent fungi of medically important genera from cockroaches from a hospital and a residential area [35]. The finding of the present study also showed that *Aspergillus niger* (70.97%) was the most common species isolated from cockroaches. Moreover, *A. flavus* and *A. fumigatus* were the most frequently recovered species from cockroaches [31]. *Aspergillus niger* was significantly more frequent in the residential area and the hospital [24]. In a study in the hospital environments in the Ahvaz city, 28 fungal species were isolated from adult houseflies. The main fungi isolated were *Aspergillus* spp. (67.4%), *Penicillium* sp. (11.6%), *Mucorales* sp. (11%), *Candida* spp. (10.5%), and *Rhodotorula* sp. (8.4%) [35].

In the present study, from the external surface of the cockroaches studied, *Aspergillus niger*, *Penicillium*, *Mucor*, and *Rhizopus* molds, from the cockroach, *Aspergillus niger* molds, and from the gastrointestinal tract of cockroaches, *Aspergillus niger* and *Penicillium*, were isolated. *Candida glabrata* were isolated from the haemocoel, the gastrointestinal tract, and the outer surface, respectively, and 2, 12, and 9 cases of *Candida krusei* were also isolated, respectively. The chromium agar assay was used for the identification of *Candida albicans*, *Candida tropicalis*, and *Candida krusei*, and other yeasts isolated by the chromium agar method belonged to other *Candida* species or other yeasts. Mirhendi and colleagues identified candidate strains isolated from patient samples and standard strains of *Candida* by culturing on the *Candida* chromium agar medium and showed that the results were in good agreement with the PCR_RFLP molecular assay [26].

We have displayed that American cockroaches transport a great number of species of medically significant fungi in their digestive system, incriminated as significant agents in nosocomial infections. Hospital-acquired fungal infections are considered consequential causes of morbidity in immune-compromised individuals, especially those remaining in the hospital for a long period [36]. The results of the 2003 phelan study showed that the German cockroach also played a very important role in the transmission of pathogens in hospitals [37]. Cockroaches living near human environments were significant vectors of etiological agents and all groups of possible pathogens such as protozoans, bacteria, helminths, and viruses. Various bacteria universally associated with these insects are recognized to inure diarrhea, dysentery, and food intoxication in humans. In the present study, *Aspergillus niger* and four species of *Candida* (*Candida kudriavzevii*, *Candida kluyveri*, *Candida glabrata*, and *Candida viswanathii*) were isolated from cockroaches. *Aspergillus niger* molds were isolated from the studied strains alone, and *Aspergillus niger* and *Penicillium* were isolated from their gastrointestinal tract. Finally, all four *Candida* species were isolated from the haemocoel and the gastrointestinal tract and the outer surface of the cockroaches. As mentioned, different species of fungi in this study and other similar studies have been isolated from cockroaches. In the present study, for the first time, four yeast strains (*Candida kudriavzevii*, *Candida kluyveri*, *Candida glabrata*, and *Candida viswanathii*) not isolated in previous studies were isolated. It is certain that a high contamination of cockroaches with pathogens can cause fungal epidemics in hospitals. Given the importance of pathogenic fungi in cockroaches, paying attention to public health and the need to control different ways of controlling them in our hospitals and medical centers are necessary [30]. Each hospital has a good prognosis for the fungal diseases in that hospital and the surrounding areas, which are important for the timely diagnosis, treatment, and control of patients. Since all of these hospitals had infectious wards, occasional reports of infection sent to researchers. Sometimes, a lot of people from the city are infected with these pathogens, and especially, they lived around these hospitals. So, the selection of these hospitals and sampling of American cockroaches can be generalized to the whole city. Of course, the results of this research will make people and officials be aware and will make it easier and more practical to control these disease vectors. Zahraei-Ramazani et al. [23] used various chemical and physical methods to control American cockroaches (*Periplaneta americana*) inside manholes of the municipal sewage disposal system in Esfahan city, Central Iran, and Esfahan's urban internetwork [23]. Their results showed that almost all of the products (excluding boric acid with bait formulation) resulted in appropriate control within one month of application. The appropriate products for chemical control of cockroaches were chlorpyrifos 5% emulsifiable concentrate (EC), diazinon 5% (EC), diazinon 0.05% (EC), and cypermethrin 5% fog. These pesticides achieved an optimal reduction of population, providing more than 90% control of cockroaches for five consecutive months. They concluded that the emulsifiable concentrates and fog formulations were more successful compared to other methods, as they can penetrate deep into the hiding places of cockroaches [23].

Therefore, factors such as the spread of fungal diseases in the city and the dispersal of hospitals, especially hospitals with infectious diseases, as well as the equipping of cities with a sewage sanitation network and related manholes that are installed and dispersed for inspection inside the municipal sewage disposal system, move cockroaches from hospitals to residential and public places and transmit fungal diseases. Therefore, performing various methods to control cockroaches in the urban space network before the invasion of cockroaches from inside the manholes into places and doing and observing hygienic points by people that do not prevent cockroaches from entering their residential areas are recommended. And, the control of cockroaches in hospitals is especially recommended.

## 5. Conclusion

In this study, yeasts were isolated from American cockroaches in Esfahan. High contamination of cockroaches with pathogens can cause fungal epidemics in hospitals and in the surrounding areas. Based on the results of this study, *Aspergillus niger*, *Penicillium*, *Mucor*, *Rhizopus*, *Candida glabrata*, and *Candida krusei* molds were isolated from Esfahan cockroaches. Of course, the results of this research will make people and officials be aware and will make it easier and more practical to control these diseases. Due to the importance of the pathogenicity of the abovementioned fungi, it is necessary to pay attention to public health and to eliminate these insects. On the other hand, awareness of the common fungi in each region and each hospital provides a good prognosis for fungal diseases in that hospital, which is important for timely diagnosis and treatment of patients.

## Figures and Tables

**Table 1:** The cumulative frequency distribution of different fungal species isolated on the basis of fungal isolation from 55 American cockroaches, Esfahan, Iran, 2016–2017.

Row	Fungi species	American cockroach (number + percentage)	
		Number	Percentage
1	*Aspergillus niger*	22	23.66
2	*Rhizopus*	2	2.15
3	*Penicillium*	4	4.30
4	*Mucor*	3	3.22
5	*Candida glabrata*	17	18.28
6	*Candida krusei* (*Candida kudriavzevii*)	23	24.73
7	Yeast (other yeast) (*Candida viswanathii* + *Candida kluyveri*)	22	23.66
8	Total number	93	100

**Table 2:** The cumulative frequency distribution of different fungal species isolated on the basis of fungal isolation from 55 American cockroaches collected from hospital sewage, Esfahan, Iran, 2016–2017.

Row	Fungi isolated	Place of fungi isolated from cockroaches			Total	
		Exterior surface no. (%)	Digestive system no. (%)	Haemocoel no. (%)	Numb	Per
1	*Aspergillus niger*	11 (50)	10 (45.45)	1 (4.55)	22	100
2	*Rhizopus*	2 (100)	—	—	2	100
3	*Penicillium*	3 (75)	1 (25)	—	4	100
4	*Mucor*	3 (100)	—	—	3	100
5	*Candida glabrata*	7 (41.18)	9 (52.94)	1 (5.88)	17	100
6	*Candida krusei (Candida kudriavzevii)*	9 (39.13)	12 (52.17)	2 (8.70)	23	100
7	Yeast (other yeast*) (Candida viswanathii* + *Candida kluyveri)*	6 (27.27)	11 (50)	5 (22.73)	22	100
	Total number	41	43	9	93	100

## Data Availability

The data used to support the findings of this study are available from the corresponding author upon request.
